# Comparing the Factors Correlated with Tuberculosis-Specific and Non-Tuberculosis-Specific Deaths in Different Age Groups among Tuberculosis-Related Deaths in Taiwan

**DOI:** 10.1371/journal.pone.0118929

**Published:** 2015-03-03

**Authors:** Yi-Chun Wu, Hsiu-Yun Lo, Shiang-Lin Yang, Da-Chen Chu, Pesus Chou

**Affiliations:** 1 Centers for Disease Control, Ministry of Health and Welfare, Taipei, Taiwan; 2 Community Medicine Research Center and Institute of Public Health, National Yang-Ming University, Taipei, Taiwan; 3 Department of Emergency Medicine, Taipei City Hospital, Taipei, Taiwan; Universidad Nacional de La Plata., ARGENTINA

## Abstract

**Background:**

Nearly 20% of tuberculosis (TB) patients die within one year, and TB-related mortality rates remain high in Taiwan. The study aimed to identify factors correlated with TB-specific deaths versus non-TB-specific deaths in different age groups among TB-related mortalities.

**Methods:**

A retrospective cohort study was conducted from 2006-2008 with newly registered TB patients receiving follow-up for 1 year. The national TB database from the Taiwan-CDC was linked with the National Vital Registry System and the National Health Insurance database. A chi-squared test and logistic regression were used to analyse the correlated factors related to TB-specific and non-TB-specific deaths in different age groups.

**Results:**

Elderly age (odds ratio [OR] 2.68-8.09), Eastern residence (OR 2.01), positive sputum bacteriology (OR 2.54), abnormal chest X-ray (OR 2.28), and comorbidity with chronic kidney disease (OR 2.35), stroke (OR 1.74) or chronic liver disease (OR 1.29) were most likely to be the cause of TB-specific deaths, whereas cancer (OR 0.79) was less likely to be implicated. For non-TB-specific deaths in patients younger than 65 years of age, male sex (OR 2.04) and comorbidity with HIV (OR 5.92), chronic kidney disease (OR 8.02), stroke (OR 3.75), cancer (OR 9.79), chronic liver disease (OR 2.71) or diabetes mellitus (OR 1.38) were risk factors.

**Conclusions:**

Different factors correlated with TB-specific deaths compared with non-TB-specific deaths, and the impact of comorbidities gradually decreased as age increased. To reduce TB-specific mortality, special consideration for TB patients with old age, Eastern residence, positive sputum bacteriology and comorbidity with chronic kidney disease or stroke is crucial. In particular, Eastern residence increased the risk of TB-specific death in all age groups. In terms of TB deaths among patients younger than 65 years of age, patients with HIV, chronic kidney disease or cancer had a 6-10 times increased risk of non-TB-specific deaths.

## Introduction

The World Health Organization (WHO) estimated that one-third of the world population is infected with tuberculosis (TB); in 2012, 8.6 million people were ill with TB, and 1.3 million deaths from TB were noted [1]. TB remains an important issue in the world. Reducing deaths is one of the major goals of TB control [2]; this goal is a component of the United Nations (UN) Millennium Development Goal, namely to halve TB prevalence and death rates by 2015 [3].

In Taiwan, the TB incidence rate has decreased from 73 to 53 per 100,000 individuals, and new cases have decreased from 16,748 to 12,338 per year (2005–2012). The TB mortality rate has decreased from 4.3 to 2.7 per 100,000 individuals, and the number of patients who died from TB decreased from 970 to 626 (2005–2012). However, approximately 2300 TB-related deaths are reported every year (nearly 20% of newly registered cases) [4]. The WHO reported that the average global death rate for new smear-positive cases is approximately 4% (2–8%); for retreatment cases, the global average death rate is approximately 7% (3–11%) [5]. In Taiwan, the death rate of TB patients is approximately 20% during the 12-month follow-up treatment outcome [4]. Another study in Taiwan reported that 16.5% of TB patients died within one year; among these deaths, 4% were directly attributed to TB, whereas the remaining 12.5% were due to other causes [6]. Therefore, both TB-specific deaths and non-TB-specific death rates remain high in Taiwan. TB control has greatly improved in recent years; however, compared with other developed countries where mortality rates are less than 1.0 per 100,000 individuals [5], Taiwan still has a long path ahead.

Some studies have indicated that old age, sex, smoking, poor nutrition, unemployment, alcoholism, delayed diagnosis, interrupted anti-TB therapy, delayed treatment, lung cavities, positive sputum smear, HIV, and chronic disease co-morbidities are factors associated with TB deaths [6–19]. However, we wanted to further explore the factors associated with TB deaths. Because TB mortality was calculated as death due to TB, we first focused on TB-specific deaths and attempted to identify important associated factors. This information could be used to alert physicians to these factors in an attempt to prevent more TB-specific deaths. We are also interested in comparing the differences between TB-specific deaths and non-TB-specific deaths to determine whether any differences between these two groups exist. In addition, different risk factors may be present in different age groups. Therefore, our study aimed to determine the factors correlated with TB-specific deaths and non-TB-specific deaths in different age groups and to compare the potential differences among these factors, with an overall goal of providing useful information for health authorities and health-care workers and decreasing the TB mortality rate in the future.

## Materials and Methods

A retrospective cohort study was conducted from 2006–2008 with newly registered TB patients receiving follow-up for 1 year. Three data resources, including (1) the National TB database from the Taiwan-CDC, (2) the National Vital Registry System from the Ministry of Health and Welfare and (3) claim data from the National Health Insurance (NHI), were linked for further analysis.

A newly diagnosed TB case was defined as bacteriologically confirmed or clinically diagnosed TB; patients who had previously received TB treatment were excluded. TB patient deaths that occurred within the one-year follow-up period were considered TB-related deaths. TB-specific deaths included TB patients in whom TB was cited as the cause of death (ICD-9 010–018), and non-TB-specific deaths included TB patients in whom TB was not cited as the cause of death (excluded ICD-9 010–018).

In total, 33,851 cases met our study’s cohort definition. In total, 5,584 (16.5%) patients died; 1,360 (4.0%) of these patients were classified as TB-specific deaths, and 4,224 (12.5%) were non-TB-specific deaths.

Age, sex, chest X-ray (CXR) results (negative, positive or unknown/not performed), initial sputum smear results (negative, positive or unknown/not performed), initial sputum culture results (negative, positive or unknown/not performed), initial sputum bacteriology result (positive: either smear or culture positive; negative: otherwise), residence (Northern, Central, Southern, Eastern Taiwan) and TB type (pulmonary TB: pulmonary TB lesions exist; extra-pulmonary TB: only extra-pulmonary TB lesions) were obtained from the National TB database. Information on the deaths was obtained from the National Vital Registry System. The co-morbidities data were collected from the NHI claim data. We defined co-morbidities as patients in the NHI claim data who were hospitalized more than once or who visited outpatient services more than twice within a year before joining the TB registry. The following six chronic illnesses were included: diabetes mellitus (DM) (ICD-9 250), stroke (ICD-9 430–438), cancer (ICD-9 140–208), chronic kidney disease (CKD) (ICD-9 585), chronic liver disease and cirrhosis (liver disease) (ICD-9 571), and HIV infection (ICD-9 042). Data were analyzed using SAS 9.2. A chi-squared test was used to compare the differences in demographic factors, X-ray and sputum results, residence area and co-morbidities between TB-specific deaths and non-TB-specific deaths with a significance threshold set at P < 0.01 (two-tailed). Multiple logistic regression analysis was used to identify the factors that correlated with death. In the regression model, the study population consisted of newly registered TB patients from 2006–2008. The dependent variables were TB-specific deaths and non-TB-specific deaths, and the independent variables were age, sex, residence (Eastern, non-Eastern), CXR results, initial sputum bacteriology results, TB type and co-morbidities (DM, stroke, cancer, CKD, HIV and liver disease). Because the majority of our TB patients were elderly, we divided the study population into three age groups (younger than 65 years, 65–74 years old and 75 years of age and older). Univariate logistic regression was used to analyse the odds ratio (OR) for the above variables in terms of TB-specific versus non-TB-specific deaths in the three age groups. To further investigate comorbidity, a multiple logistic regression model was also used to identify the factors associated with the different age groups in terms of TB-specific deaths and non-TB-specific deaths. To narrow our focus on the impact of different comorbidities, we only used sex, residence and co-morbidities as the independent variables.

The study was approved by the Taiwan-CDC Review Board (IRB No. 102019). The patient records were anonymized and de-identified prior to the analysis.

## Results

### The characteristics of TB-specific deaths and non-TB-specific deaths

Generally, male sex, old age, abnormal CXR result, initial positive sputum bacteriology, pulmonary TB type and non-Eastern residence were the major characteristics of total TB deaths. Various characteristics significantly differed (P < 0.01) between these two groups, including age, residence, CXR, sputum bacteriology and comorbidities with DM, cancer, stroke, chronic liver disease and CKD, but excluding sex and TB type. When comparing these two groups, advanced age, Eastern residence, abnormal CXR, positive sputum bacteriology and absence of comorbidity were more frequently associated with TB-specific deaths. The above details are presented in Table 1.

**Table 1:** The Characteristics of TB-Specific Deaths and Non-TB-Specific Deaths.

Variables		Total deaths	(%)	TB-specific		Non-TB-specific		p-value
				N	(rate %)	N	(rate %)	
Total		5,584		1,360	24.4	4,224	75.4	
**Age**								< 0.001
	0–44	195	3.5	46	23.6	149	76.4	
	45–64	770	13.8	167	21.7	603	78.3	
	65–74	1,035	18.5	207	20.0	828	80.0	
	≥ 75	3,584	64.2	940	26.2	2,644	73.8	
**Sex**								0.75
	Male	4,165	74.6	1,010	24.2	3,155	75.8	
	Female	1,419	25.4	350	24.7	1,069	75.3	
**Residence**								< 0.01
	Northern	2,138	38.3	516	24.1	1,622	75.9	
	Central	1,128	20.2	278	24.6	850	75.4	
	Southern	2,067	37.0	481	23.3	1,586	76.7	
	Eastern	251	4.5	85	33.9	166	66.1	
**Initial sputum bacteriology**								< 0.001
	Negative	873	15.6	170	19.5	703	80.5	
	Positive	4,711	84.4	1,190	25.3	3,521	74.7	
**Initial CXR**								< 0.001
	Normal	249	4.5	34	13.7	215	86.3	
	Abnormal	5,148	92.2	1,282	24.9	3,866	75.1	
	Unknown/not performed	187	3.3	44	23.5	143	76.5	
**Type of TB**								0.25
	Extrapulmonary	134	2.4	27	20.1	107	79.9	
	Pulmonary	5,450	97.6	1,333	24.5	4,117	75.5	
**Comorbidity**								
DM								< 0.001
	Yes	1,645	29.5	351	21.3	1,294	78.7	
	No	3,939	70.5	1,009	25.6	2,930	74.4	
Cancer								< 0.001
	Yes	1,451	26.0	158	10.9	1,293	89.1	
	No	4,133	74.0	1,202	29.1	2,931	70.1	
Stroke								0.01
	Yes	1,612	28.9	354	22.0	1,258	78.0	
	No	3,972	71.1	1,006	25.3	2,966	74.7	
Liver disease								< 0.001
	Yes	646	11.6	108	16.7	538	83.3	
	No	5,120	91.7	1,252	24.5	3,868	75.5	
CKD								< 0.001
	Yes	721	12.9	141	19.6	580	80.4	
	Yes	4,863	87.1	1,219	25.1	3,644	74.9	
HIV								0.09
	Yes	28	0.5	3	10.7	25	89.3	
	No	5,556	99.5	1,357	24.4	4,199	75.6	

Initial sputum bacteriology (positive, either initial smear or culture positive; otherwise, negative)

Yes: with disease; No: without disease

Stroke: ICD-9 430–438; CKD: chronic kidney disease, ICD-9 585; DM: diabetes mellitus, ICD-9 250; Cancer: ICD-9 140–208; Liver disease: chronic liver disease and cirrhosis, ICD-9 571; HIV: ICD-9 042

### The predictors of TB-specific and non-TB-specific deaths

In Table 2, we observed that elderly age (OR 2.68–8.09), Eastern residence (OR 2.01, confidence interval [CI] 1.58–2.56), positive sputum bacteriology (OR 2.54, CI 2.13–3.04), abnormal CXR (OR 2.28, CI 1.51–3.43), unknown CXR (OR 3.11, CI 1.93–5.00) and co-morbidity with CKD (OR 2.35, CI 1.94–2.86), stroke (OR 1.74, CI 1.52–2.00) and chronic liver disease (OR 1.29, CI 1.05–1.59) increased the risk of TB-specific death. Patients with TB comorbid with cancer (OR 0.79, CI 0.66–0.94) were less likely to succumb to TB-specific death. No significant associations were observed between TB-specific death and sex or TB-specific death and co-morbidity with HIV or DM. However, the following factors were more frequently noted in non-TB-specific deaths: elderly age (OR 2.44–5.49), male sex (OR 1.18, CI 1.09–1.28), Eastern residence (OR 1.28, CI 1.07–1.53), positive sputum bacteriology (OR 1.85, CI 1.68–2.04), unknown CXR results (OR 1.66, CI 1.28–2.16), and co-morbidity with HIV (OR 4.73, CI 3.02–7.40), CKD (OR 3.63, CI 3.22–4.09), stroke (OR 2.49, CI 2.29–2.71), cancer (OR 3.15, CI 2.90–3.41), chronic liver disease (OR 2.00, CI 1.78–2.24) and DM (OR 1.24, CI 1.14–1.34). Different predictors were observed for TB-specific deaths versus non-TB-specific deaths.

**Table 2:** The Predictors of TB-Specific Deaths and Non-TB-Specific Deaths.

Variables		TB-specific		Non-TB-specific	
		OR (stepwise)	95% CI	OR (stepwise)	95% CI
**Age**					
	65–74 vs. < 65	2.68^*^	2.20–3.30	2.44^*^	2.18–2.72
	75+ vs. < 65	8.09^*^	6.91–9.48	5.49^*^	5.01–6.02
**Sex**					
	Male vs. Female	1.12	0.99–1.28	1.18^*^	1.09–1.28
**Residence**					
	Eastern vs. non-Eastern	2.01^*^	1.58–2.56	1.28^*^	1.07–1.53
				
**Initial sputum bacteriology**					
	Positive vs. Negative	2.54^*^	2.13–3.04	1.85^*^	1.68–2.04
**Initial CXR**					
	Abnormal vs. Normal	2.28^*^	1.51–3.43	1.04	0.86–1.25
	Unknown or not performed vs. Normal	3.11^*^	1.93–5.00	1.66^*^	1.28–2.16
**Types of TB**					
	Pulmonary vs. Extrapulmonary	0.67	0.40–1.12	1.31	0.99–1.73
**Comorbidity**					
	HIV			4.73^*^	3.02–7.40
	CKD	2.35^*^	1.94–2.86	3.63^*^	3.22–4.09
	Stroke	1.74^*^	1.52–2.00	2.49^*^	2.29–2.71
	Cancer	0.79^*^	0.66–0.94	3.15^*^	2.90–3.42
	Liver disease		1.05–1.59	2.00^*^	1.78–2.24
	DM			1.24^*^	1.14–1.34

Initial sputum bacteriology (positive, either initial smear or culture positive; otherwise, negative).

*: Reached statistically significant difference.

Stroke: ICD-9 430–438; CKD: chronic kidney disease, ICD-9 585; DM: diabetes mellitus, ICD-9 250; Cancer: ICD-9 140–208; Liver disease: chronic liver disease and cirrhosis, ICD-9 571; HIV: ICD-9 042

### Univariate analysis of TB-specific versus non-TB-specific deaths in different age groups

Elderly people comprised the majority of TB deaths. Age was the most significant factor associated with both TB-specific deaths and non-TB-specific deaths (as shown in Tables 1 and 2), as well as the different associated factors observed in these two groups; therefore, the study further divided the study population into three age groups (younger than 65 years of age, 65–74 years of age, and 75 years of age and older) and analyzed the OR for each variable associated with the TB-specific deaths versus the non-TB-specific deaths in the 3 age groups.

Table 3 (i.e., univariate analysis) shows that in the age group younger than 65 years, Eastern residence (OR 2.24, CI 1.34–3.73), abnormal CXR (OR 2.94, CI 1.04–8.32), and positive sputum bacteriology (OR 2.10, CI 1.35–3.28) were risk factors for TB-specific versus non-TB-specific deaths, but comorbidities with CKD, cancer and chronic liver disease were less likely to be risk factors. For patients aged 65–74 years, abnormal CXR (OR 2.56, CI 1.01–6.52) was a risk factor for TB-specific versus non-TB-specific deaths, whereas comorbidities with CKD, cancer and chronic liver disease were less likely to be risk factors. For patients 75 years of age and older, Eastern residence (OR 1.48, CI 1.03–2.11) and abnormal CXR (OR 1.83, CI 1.19–2.84) were risk factors, but comorbidities with stroke, cancer, and DM were less likely to be risk factors.

**Table 3:** Univariate Analysis of TB-Specific Deaths versus Non-TB-Specific Deaths in Different Age Groups.

		Younger than 65 y/o		65–74 y/o		75 y/o and older	
		No	OR (95% CI)	No	OR (95% CI)	No	OR (95% CI)
**Total**		965		1,035		3,584	
**Sex**							
	Male	787	1.05 (0.71–1.57)	800	1.15 (0.79–1.67)	2,578	0.96 (0.82–1.14)
**Residence**							
	Eastern	70	2.24 (1.34–3.73)^*^	40	1.55 (0.76–3.15)	141	1.48 (1.03–2.11)^*^
**Initial CXR**							
	Abnormal	897	2.94 (1.04–8.32)^*^	945	2.56 (1.01–6.52)^*^	3,306	1.83 (1.19–2.84)^*^
	Unknown/not performed	24	2.63 (0.63–10.92)	36	1.96 (0.55–6.98)	127	1.77 (0.99–3.17)
**Initial sputum bacteriology**							
	Positive	769	2.10 (1.35–3.28)^*^	861	1.44 (0.93–2.24)	3,081	1.20 (0.96–1.50)
**Types of TB**							
	Pulmonary	934	1.49 (0.57–3.93)	1,008	6.68 (0.90–49.47)	3,508	0.93 (0.56–1.55)
**Comorbidity**							
	HIV	26	0.45(0.14–1.52)	1	NA	1	NA
	CKD	139	0.52 (0.31–0.86)^*^	154	0.55 (0.34–0.91)^*^	428	0.87 (0.68–1.10)
	Stroke	126	0.96 (0.61–1.51)	271	0.72 (0.50–1.03)	1,215	0.79 (0.67–0.93)^*^
	Cancer	334	0.10 (0.06–0.18)^*^	338	0.38 (0.26–0.56)^*^	779	0.37 (0.30–0.46)^*^
	Liver	210	0.45 (0.29–0.70)^*^	168	0.56 (0.35–0.90)^*^	268	0.78 (0.58–1.05)
	DM	287	0.80 (0.57–1.13)	355	0.83 (0.60–1.15)	1,003	0.79 (0.67–0.94)^*^

*: Reached statistically significant difference.

### Analysis of comorbidity on TB-related deaths in different ages

Regarding the analysis of comorbidity (Table 4), for patients younger than 65 years of age, male sex (OR 2.36, CI 1.65–3.36), Eastern residence (OR 2.37, CI 1.56–3.61), and comorbidities with CKD (OR 3.73, CI 2.27–6.12) and stroke (OR 4.02, CI 2.63–6.13) were more likely to yield TB-specific deaths; however, male sex (OR 2.04, CI 1.67–2.49) and co-morbidities with HIV (OR 5.92, CI 3.65–9.62), CKD (OR 8.02, CI 6.22–10.48), stroke (OR 3.75, CI 2.88–4.88), cancer (OR 9.79, CI 8.28–11.58), chronic liver disease (OR 2.71, CI 2.23–3.31) and DM (OR 1.38, CI 1.15–1.65) were more likely to yield non-TB-specific deaths. For patients 65–74 years of age, male sex (OR 1.53, CI 1.08–2.15), Eastern residence (OR 2.00, CI 1.07–3.76), CKD (OR 1.95, CI 1.21–3.15) and stroke (OR 1.95, CI 1.38–2.76) were risk factors for TB-specific deaths; male sex (OR 1.33, CI 1.11–1.60), CKD (OR 3.75, CI 2.96–4.75), stroke (OR 3.15, CI 2.62–3.79), cancer (OR 3.38, CI 2.86–4.01) and chronic liver disease (OR 2.17, CI 1.75–2.69) tended to be related to non-TB-specific deaths. For patients 75 years of age and older, Eastern residence (OR 1.95, CI 1.41–2.70), CKD (OR 2.02, CI 1.61–2.54) and stroke (OR 1.54, CI 1.33–1.79) tended to be related to TB-specific deaths, but cancer (OR 0.68, CI 0.55–0.84) was unlikely to be related; Eastern residence (OR 1.44, CI 1.11–1.87) and comorbidities with CKD (OR 2.32, CI 1.98–2.71), stroke (OR 2.04, CI 1.84–2.25), cancer (OR 1.88, CI 1.68–2.10), chronic liver disease (OR 1.34, CI 1.12–1.60), and DM (OR 1.22, CI 1.10–1.35) tended to be related to non-TB-specific deaths.

**Table 4:** Stepwise Logistic Regression Analysis of Comorbidity on TB-Related Deaths in Different Age Groups

		Younger than 65 y/o OR (95% CI)		65–74 y/o OR (95% CI)		75 y/o and older OR (95% CI)	
		TB-specific	Non-TB-specific	TB-specific	Non-TB-specific	TB-specific	Non-TB-specific
**Sex**							
	Male	2.36 (1.65–3.36)^*^	2.04 (1.67–2.49)^*^	1.53 (1.08–2.15)^*^	1.33 (1.11–1.60)^*^		
**Residence**							
	Eastern	2.37 (1.56–3.61)^*^		2.00 (1.07–3.76)^*^		1.95 (1.41–2.70)^*^	1.44 (1.11–1.87)^*^
**Comorbidity**							
	HIV		5.92 (3.65–9.62)^*^				
	CKD	3.73 (2.27–6.12)^*^	8.02 (6.22–10.34)^*^	1.95 (1.21–3.15)^*^	3.75 (2.96–4.75)^*^	2.02 (1.61–2.54)^*^	2.32 (1.98–2.71)^*^
	Stroke	4.02 (2.63–6.13)^*^	3.75 (2.88–4.88)^*^	1.95 (1.38–2.76)^*^	3.15 (2.62–3.79)^*^	1.54 (1.33–1.79)^*^	2.04 (1.84–2.25)^*^
	Cancer		9.79 (8.28–11.58)^*^		3.38 (2.86–4.01)^*^	0.68 (0.55–0.84)^*^	1.88 (1.68–2.10)^*^
	Liver	1.39 (0.92–2.11)	2.71 (2.23–3.31)^*^		2.17 (1.75–2.69)^*^		1.34 (1.12–1.60)^*^
	DM		1.38 (1.15–1.65)^*^				1.22 (1.10–1.35)^*^

*: Reached statistically significant difference.

The impact of co-morbidities was reduced in TB-specific deaths, whereas Eastern residence displayed a greater effect. Eastern residence was the risk factor for all age TB-specific deaths, but it was a risk factor only for non-TB-specific deaths in the 75 years of age and older group. The impacts of co-morbidities and male sex were gradually reduced when age increased in both TB-specific and non-TB-specific deaths.

## Discussion

The cause of death as registered by physicians is often subjective. TB was occasionally recorded as the underlying cause of death in patients with TB despite the involvement of other factors. To account for this discrepancy, the Taiwan-CDC initiated a review of TB-related death certificates by TB experts beginning in 2002. Therefore, an abrupt decrease in the TB mortality rate occurred from 2003 to 2004 [20]. Since then, the TB mortality rate has slowly decreased. However, in 2012, the TB (TB-specific) mortality rate remained at 2.7 per 100,000 individuals in Taiwan. A considerable amount of work must be performed to achieve the goal of a TB mortality rate less than 1.0 per 100,000 individuals. In this study, we aimed at identifying factors associated with TB-specific and non-TB-specific deaths to aid in the reduction of the TB mortality rate in the future.

We observed that the characteristics and factors correlated with TB-specific and non-TB-specific deaths were different (Tables 1 and 2). Positive sputum bacteriology was a risk factor for TB-specific deaths and that the risk was higher than for non-TB-specific deaths (Table 2). These findings are consistent with our experience wherein patients typically have positive sputum tests with severe TB cases, and these patients would easily be classified as death due to TB. Another prominent feature was the difference in co-morbidity with cancer between these two study groups. In the regression model, we also observed that patients with cancer were less likely to have a TB-specific death (Table 2). This finding might be attributed to the fact that TB patients with cancer often suffer from complicated side effects related to chemotherapy or radiotherapy for anti-cancer treatment or are experiencing the terminal stages of cancer.

Male sex, old age, positive sputum bacteriology, Eastern residence and co-morbidities with HIV, CKD, stroke, cancer, chronic liver disease and DM were more likely to result in non-TB-specific deaths (Table 2) [6]. The effects of old age, Eastern residence, positive sputum bacteriology, abnormal CXR and unknown or unperformed CXR were also more prominent in TB-specific deaths (Table 2). In particular, Eastern residence was strongly correlated with TB-specific deaths, possibly because there are less medical resources in this region of Taiwan compared with the less mountainous Western areas. Previous studies reveal that TB incidence and mortality are quite high in the mountainous areas and regions of Eastern Taiwan [21, 22].

We demonstrated that the co-morbidities had a relatively minor effect on TB-specific deaths. CKD and stroke were the factors that correlated with TB-specific deaths, and patients with cancer were less likely to experience a TB-specific death. Previous studies revealed that TB patients with co-morbidities potentially die more easily. In our study, co-morbidities were easily determined because they were often listed as the primary cause of death. TB is a curable disease, so it is generally considered unlikely for a patient to die from TB in the absence of severe or complicated chronic disease. In our study, we found that TB patients with CKD, stroke or chronic liver disease were likely to experience a TB-specific death. This finding reveals that even if a patient had CKD, stroke or chronic liver disease, physicians still made the diagnosis of mortality due to TB. We assumed that these chronic diseases (CKD, chronic liver disease or stroke) were stable and did not serve as the cause of death, but TB treatments were perhaps unsuccessful in these patients. Further investigation is needed to clarify the reasons. We assumed that patients with CKD, stroke or chronic liver disease might require more attention for further treatment or evaluation of drug effects to prevent TB-specific deaths.

The study also found that in the different age groups, the factors associated with TB-specific deaths differed from those associated with non-TB-specific deaths. Univariate analysis demonstrated that abnormal CXR was a risk factor for all age groups for TB-specific deaths versus non-TB-specific deaths, but the risk decreased as patient age increased (Table 3). Cancer comorbidity was less likely to be associated with TB-specific deaths compared with non-TB-specific deaths for all age groups. CKD was less likely to be associated with TB-specific deaths (compared with non-TB-specific deaths) for patients younger than 75 years. Stroke and DM were less likely to be associated with TB-specific deaths (compared with non-TB-specific deaths) for patients 75 years of age and older, and the association with chronic liver disease was weaker for patients younger than 75 years of age (Table 3).

When considering sex, residence and comorbidities, Eastern residence and co-morbidity with CKD and stroke were risk factors for TB-specific deaths in the three age groups, but male sex was only a risk factor for patients younger than 65 years of age and 65–74 years of age; cancer was less likely to be a risk factor only for patients 75 years of age and older (Table 4). For non-TB-specific deaths, male sex was a risk factor, except for patients 75 years of age and older; CKD, stroke, cancer, and chronic liver disease were risk factors for all age groups, but HIV tended to be a risk factor for patients younger than 65 years of age; DM was a risk factor for patients younger than 65 years of age and 75 years of age and older (Table 4). However, for both TB-specific deaths and non-TB-specific deaths, the impact of co-morbidities and male sex gradually decreased as the patient age increased (Table 4). The underlying causes for the variations of the factors associated with different age groups require further investigation. However, the study results provide more detailed and useful information for different TB control programs for different age groups.

In conclusion, this study revealed that the characteristics and factors correlated with TB-specific deaths and non-TB-specific deaths differ and that comorbidities affect different age groups differently. The comorbidities displayed less of an effect in TB-specific deaths, whereas age and Eastern residence had a greater effect. The impacts of comorbidities and male sex gradually reduced as age increased for both TB-specific deaths and non-TB-specific deaths. To reduce the TB (TB-specific) mortality rate, it is crucial for more attention to be paid to elderly patients and patients with positive sputum bacteriology, Eastern residence and CKD, chronic liver disease and stroke comorbidities. In particular, residing in Eastern Taiwan was a risk factor for TB-specific death in all age groups. More medical resources and public health efforts may be necessary for the Eastern region of Taiwan. For TB deaths in the younger than 65 years old age group, patients with HIV, CKD or cancer had a 6–10 times greater risk of non-TB-specific deaths.
